# Assessment of anti-depressant effect of nelumbinis semen on rats under chronic mild stress and its subchronic oral toxicity in rats and beagle dogs

**DOI:** 10.1186/1472-6882-12-68

**Published:** 2012-05-28

**Authors:** Hwan-Suck Chung, Hye Jeong Lee, Insop Shim, Hyunsu Bae

**Affiliations:** 1 Department of Physiology, College of Oriental Medicine, Kyung Hee University, #1 Hoeki-Dong, Dongdaemoon-gu, Seoul, 130-701, Republic of Korea; 2 Acupuncture & Meridian Science Research Center, College of Oriental Medicine, Kyung Hee University, Seoul, 130-701, Republic of Korea

**Keywords:** Nelumbinis semen, Depression, Toxicity, Open field test

## Abstract

**Background:**

Previously, we examined the antidepressant effects of Nelumbinis Semen (NS). In this study, we assessed the anti-depressant effects of NS in the forced swimming test and chronic mild stress (CMS) models of depression and its oral toxicity in rats and dogs.

**Methods:**

In the forced swimming test, NS was intraperitoneally injected before 24 h, 5 h and 1 h of forced swimming test. And the rats were forced to swim for 5 min, the duration of immobility was observed. In CMS models, animals were exposed to a variety of CMS for 8 weeks in order to induce depression-like symptoms. They were treated with NS for the last four weeks of the 8-week CMS and then an open field test was conducted. The anti-depression effects were evaluated based on a measured index, which consisted of visiting counts, start latency, rearing number and grooming time. In the toxicological studies, NS was administered to rats by gavages for 13 weeks at doses of 0, 500, 1000, and 2000 mg/kg/day. To assess the toxicity of NS in beagle dogs, NS was administered orally for 28 days at doses of 0, 500, 1000, 2000 and 4000 mg/kg/day.

**Results:**

400 mg/kg of NS had the lowest immobility times in forced swimming test. And NS significantly reversed the decreased visiting counts, rearing number and grooming time caused by CMS. In addition, NS treatment significantly decreased the start latency. No treatment-related toxicity was detected during 13 weeks administration in rats and 28 days administration in dogs.

**Conclusions:**

Based on the results of this study and previous reports that have examined the anti-depressive effects of NS, NS holds great promise for use in the treatment of depression without causing any adverse effects or toxicities.

## Backgrounds

*Nelumbo nucifera* is a perennial aquatic plant grown and consumed all over the world, especially in India and South East Asia. Nelumbinis Semen (NS, the seeds of *Nelumbo nucifera*) or lotus seed is a traditional medicine that has been used for hundreds of years in East-Asia to treat insomnia, anxiety and women's post-menstrual-pause depression [1]. NS contains alkyl 4-hydroxybenzoates [2], some bisbenzylisoquinoline alkaloids [3-5], benzylisoquinoline alkaloids [4], aporphine and proaporphine alkaloids[6]. In previous studies, we found that this herbal medicine exerted an antidepressant-like effect in rats based on the forced swim test. The results of that study suggested that NS could increase local cholinergic and dopaminergic or norephinergic neurotransmission via activation of cAMP formation in the hippocampus and pre-frontal cortex [7,8]. Natural products have long been used in traditional medicine to treat inflammation and other inflammation-related diseases, and the raw materials of these products are often used to develop new drugs [9,10].

Many factors contribute to the appeal of herbal medicine and herbs have been used to both treat and prevent diseases. Herbal treatments are considered safe because they are a natural, gentle, and harmless alternative to conventional medicine. However, there is a lack of evidence related with the toxicology of herbal medicines and some people insist that herbal medicines are toxic to the liver. Although the root and leaf of *Nelumbo nucifera* has been used in health food or tea, the safety of NS had not been previously established, which is a needed before this herbal medicine can be used as a new drug.

The aim of this study was to investigate the anti-depressive effects and the toxicity of NS after repeated oral administration.

## Methods

### Animal

In the pharmacological study, 6 week old male Wister rats, weighing 180 ~ 200 g, were supplied by Jung-Ang Experimental Animal Center (Seoul, Korea). The rats were allowed free access to their diets and tap water, except during the scheduled CMS. The rats were adapted to this environment for 1 week prior to the experiments. All procedures involving the use of the animals were approved by the Institutional Animal Care and Use Committee of Kyung Hee University and were carried out in accordance with the ethical guidelines.

In the 13-week oral toxicity study, 48 male and 48 female Sprague–Dawley (SD) specific pathogen free (SPF) rats 5 weeks old were obtained from Koatec Inc (Gyeonggi-do, Korea). For the 4-week oral toxicity study in dogs, 6 male and 6 female Beagle dogs were received from Beijing Marshall Biotechnology Company (Beijing, China). The dogs were 6 months old and weighed 7.88–8.58 kg upon initiation of treatment. They were housed individually in stainless-steel cages in a controlled environment (temperature 23 ± 3 °C, humidity 55 ± 15%, 12 h light/12 h dark cycles). Ventilation was given 10 to 20 times/h and a quantitative pellet diet was provided at a fixed time each day. During a 2-week acclimatization period, parameters including body weight, temperature, appetite and performance of the dogs were observed and recorded before treatment.

The toxicity study was conducted at the Good Laboratory Practice (GLP) institute approved by the Korea Food and Drug Administration (KFDA), in compliance with the GLP and Test Guidelines of the Organization for Economic Cooperation and Development [11] and the KFDA [12]. The study protocol was approved by the Institutional Animal Care and Use Committee (IACUC) of the institute. (accredited by AAALAC International, 2010).

### Forced swim test

Male Sprague–Dawley rats, weighed 260 to 310 g, were supplied by Korea Taconic Co., Ltd. (South Korea). The animals were allowed to adapt to lab conditions for 2 weeks prior to the commencement of the experiments. Rats were individually placed in a glass cylinder (20 cm diameter × 40 cm high) containing water (24 ± 1 °C) with a depth of 20 cm from the bottom. All rats were placed in cylinders filled with water and forced to swim for 15 min on day 1. NS dissolved in saline was intraperitoneally treated before 24 h, 5 h and 1 h of forced swimming test. On day 2, all rats were forced to swim for 5 min, and the duration of immobility was observed and measured. The immobility time was regarded as the time the mouse spent floating in the water without struggling and making only those movements necessary to keep its head above water.

### Chronic mild stress

In order to induce CMS and depression-like behavior, rats were consistently exposed to various mild stressors, such as overnight illumination, food and/or water deprivation, cage tilt and change of cage mate over an 8 week period, as described previously [7]. These stressors were changed every few hours over the 8 week period.

### Preparation of anti-depressants

The sprayed-dry extracts of Nelumbinis Semen were purchased from the Sun-Ten Pharmaceutical Company, Taiwan. *Hyperium Perforatum* and fluoxetine were used as the positive controls. The *Hyperatum Perforatum* was purchased from HBC Protocols Company (Los Angeles, CA, USA). The Hypericin concentration, which is the standard material for *Hyperatum Perforatum*, was 0.3%. This compound has been widely used as an index in animal studies [13]. Fluoxetine (Prozac), a selective serotonin reuptake inhibitor (SSRI), was purchased from Sigma Company (St. Louis, MO, USA).

### Drug administration

Nelumbinis Semen (400 mg/kg/day), *Hyperium Perforatum* (2.68 g/kg/day) or fluoxetine (10 mg/kg/day) was administered orally (Nelumbinis Semen and Hyperium Perforatum) or intraperitoneally (fluoxetine) for the last 4 weeks during the 8 week-CMS.

### Open field activity

In order to evaluate the difference between CMS exposure and drug administration, several rat behaviors were observed and required in an open field. The open field consisted of a wooden box with dimensions of 75 × 75 × 30 cm. The floor of the box was divided into 15 cm squares using lines. The open field was connected to a small box with dimensions of 15 × 15 × 15 cm, which was used as the start box where the experimental animals was placed prior to entering the open field. A vertical sliding door was installed between the start box and the open field. Rats were placed in the start box, and, after 30 sec, the sliding door was open. The time to leave the start box toward the open field, which was expressed as the start latency, was recorded. The start latency time corresponds to the time from when the door was opened to when the rat tail completely exited the start box. Immediately after the start latency, open field behaviors of rats were observed and recorded. The following behaviors were recorded over a total of 10 min: locomotion, rearing, grooming and defecation. Grooming behavior was recorded as the total time spent grooming over 10 min, and the remaining behaviors were all recorded as frequencies. Locomotor activity was recorded by counting the number of squares each rat crossed on the floor, which was divided into 25 15 × 15-cm squares, using a computer tracking system. Rearing was recorded as the total number of times each rat displayed exploratory behavior, while standing on its hind paws during 10 min. Grooming was recorded as the number of times each rat stroked its head or combed its fur with its forepaws during 10 min.

### Rat toxicity studies

The 13-week oral dose study in rats was performed to assess the general toxicity of NS in rats (n = 5/sex/dose group) at doses of 0, 500, 1000 and 2000 mg/kg following daily gavage administration. The animals were randomized by sex into one of the four dose groups. After NS was administered orally at dose levels of 0, 500, 1000 and 2000 mg/kg/day (male 5, female 5) for 13 weeks, several parameters such as mortality, clinical signs, body weight changes, food and water consumption, urinalysis, hematology and serum biochemistry examinations, necropsy findings and relative organ weights were recorded.

### Dog toxicity studies

1 male and 1 female dogs each were treated with 0, 500, 1000, 2000, and 4000 mg/kg NS, respectively, via oral administration once daily for 28 days. Body weight and average food consumption of the dogs were recorded every week. Their body temperatures were measured before and after administration. The animals were observed carefully throughout the experiment, especially the moment after administration. Objective signs including color pattern, cleanliness, behavior, food intake, urine, manure, psychic states, eye and porous channel secretions were measured and recorded every day.

### Blood analysis

All live animals were subjected to hematological tests before grouping and on the scheduled necropsy day. A portion of blood sample from the cephalic vein was collected in a serum separation tube (Vacutainer tube, SST, Gel & clot Activator). The blood was kept for 15–20 min at room temperature then centrifuged at 3,000 rpm for 10 min and the supernatant serum was used for analysis.

### Urinalysis

Before administration and two day before necropsy, the urine was examined using a test strip for urinalysis (Bayer) and an automatic urinalysis system (CliniTek 100, Bayer). The urinalysis test items were glucose, bilirubin, ketone body, specific gravity, occult blood, pH, protein, urobilinogen, nitrite and leukocyte.

### Autopsy and histopathological study

Animals were sacrificed by exsanguinations and dissected. During the process of dissecting, the color, texture and lump of parenchymatous organs were carefully examined. The color and integrity of the cavities’ mucosa were also examined. In addition, the weight of the brain, hypothyroids, lungs, heart, thymus glands, liver, spleen, kidneys, adrenal glands, prostate, testicle, and ovaries were measured and recorded. The organ-body index was calculated according to the following formula [14]: Organ-body index (%) = Wet organ weight/Body weight × 100%.

All extracted organs from all animals were fixed in a 10% neutral formaldehyde solution, except for the testes, which were fixed in Bouin’s solution.

### Statistical analysis

To compare the test groups with the control group, parametric or non-parametric multiple comparison procedures were applied All statistical analysis were performed using the commercial statistical package SPSS 10.1. In the case of continuous data, such as open field activity, body weights, food and water consumptions, hematology and serum biochemistry, and relative organ weights, data expressed with mean ± S.D were subjected to One-Way ANOVA test to assess significance. Cases that showed a significant difference were subjected to the Levene test to assess equality of variances. If homoscedasticity of the data was accepted, the Duncan’s multiple range test was applied. Otherwise, Dunnett’s *t*-test was conducted to examine the significance between the treatment groups and control. For analysis of discontinuous data such as urinalysis, the data was subjected to scale transformation, which expresses the data as the severity of the signs (Table 1).

**Table 1:** Scale transformation of urinalysis data according as the severity of the signs

Scales	GLU, KET, PRO	OB, WBC	BIL	SG	pH	URO	NIT
0	-	-	-	≤1.005	6.0	0.1	-
1	±	±	1+	1.010	6.5	1	+
2	1+	1+	2+	1.015	7.0		
3	2+	2+	3+	1.020	7.5		
4	3+	3+	4+	1.025	8.0		
5	4+			≥1.030			

GLU = Glucose, BIL = bilirubin, KET = ketone body, SG = specific gravity, OB = occult blood, pH, PRO = protein, URO = urobilinogen, NIT = nitrite and WBC = leukocyte.

For non-parametric multiple comparison, Kruskal-Wallis´ H-test was performed. Cases that showed a significant difference were analyzed by the Mann–Whitney *U*-test and Student’s *t*-test. In dog toxicity studies, statistical analysis was not carried out because each group included only 1 animal of both sexes.

## Results

### Evaluation of antidepressive activity of the NS by an animal behavioral test in a forced swimming test (FST) and a CMS model of depression

**Figure 1:**
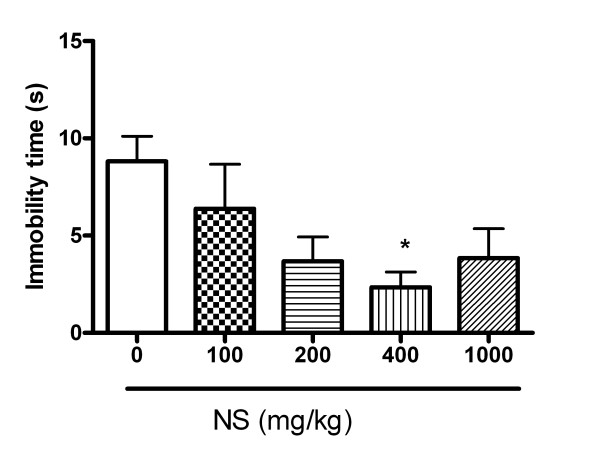
**The anti-depression effect of NS on the durations of immobility in the forced swim test.** NS or saline was intraperitoneally injected before 24 h, 5 h and 1 h of the forced swim test, rats (n = 5 in each group) were individually placed in a glass cylinder containing water. During the 5 min test, the duration of immobility was observed and measured. The immobility time was regarded as the time spent by the mouse floating in the water without struggling. *P < 0.05 compared to control.

It is known that the stressed mice exhibited a markedly depressed phenotype characterized by increased durations of immobility during the FST when compared with non-stressed mice [15]. To determine the maximal effective dose of NS in FST, we treated NS at various doses. As shown Figure 1, 400 mg/kg of NS had the lowest immobility times in FST. Based on this result, we compared the anti-depressant effect of NS with Prozac and natural St. John's wort in the open field test.

**Figure 2:**
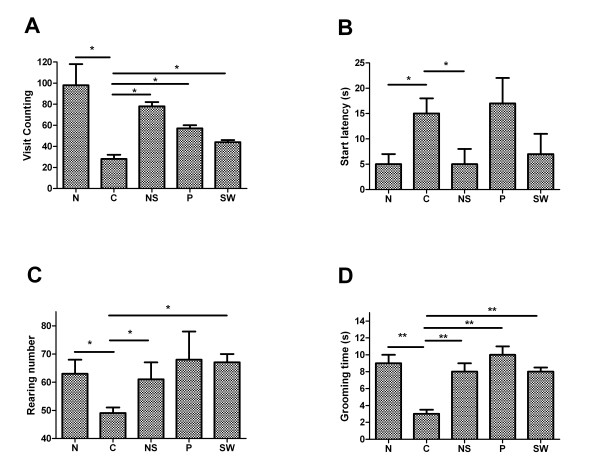
**The anti-depression effect of NS based on (A) visit counts, (B) start latency (C) rearing numbers and (D) grooming time in the open field test after antidepressant treatment during the last 4 weeks of the 8-week CMS.** N = normal group without any condition and treatment, C = control group without any treatment under CMS, NS = NS treatment group under CMS, *P* = Prozac treatment group under CMS and SW = St. John's Wort treatment group under CMS. 6 rats were used in each group. *P < 0.05 and **P < 0.01 compared to control.

After drug administration for the last four weeks of the 8-week CMS period, the total number of chambers which each animal visited (visit counts) was recorded in the open field test. As shown in Figure 2A, compared to the control group (C), the NS group displayed a significant increase in visit counts. These visit counts were greater than those of the representative conventional antidepressants, Prozac (P) and natural St. John's wort (SW), demonstrating that the NS extract produced a strong antidepressive effect. Also, these results indicate that NS administration reversed the rat activity caused by CMS-induced depression.

The effect of NS treatment on the start latency time was also measured. As shown in Figure 2B, compared to the control group (C), only the NS group displayed a significant decrease (P < 0.05) in start latency. NS administration resulted in a shortened start latency. These results indicate that the CMS-induced reduction in curiosity and the will to live was reversed by treatment with NS.

Rearing behavior in the open field was also recorded after drug administration for the last four weeks of the 8-week CMS procedure. As shown in Figure 2C, compared to the control group, the NS group displayed a significant decrease (P < 0.05) in rearing frequency. These results indicate that NS administration restored the exploratory behavior of rats when in a new environment. Thus, the CMS-induced reduction in the curiosity and exploratory behavior to neighboring environments was reversed by treatment with the NS extract.

Grooming behavior in the open field was recorded after drug administration for the last four weeks of the 8-week CMS procedure. As shown in Figure 2D, compared to the control group, the NS group displayed a significant increase (P < 0.05) in grooming time. This antidepressive effect was similar to that of the conventional antidepressant, the St. John's wort plant. These results indicate that NS administration restored the rat’s interest in itself. Thus, the CMS-induced reduction in self-interest was reversed by treatment with the NS extract.

### Subchronic 13-week oral toxicity in rat

**Figure 3:**
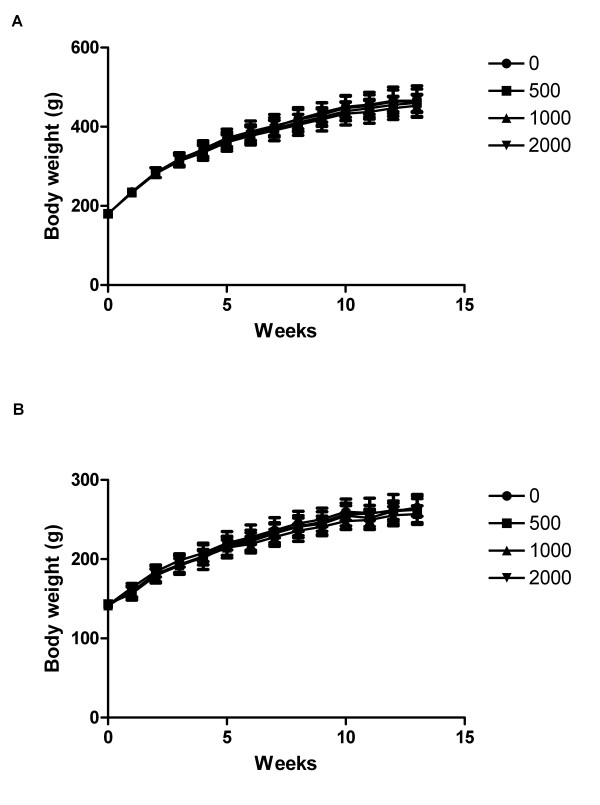
Growth curve of male (A) and female (B) rats fed NS.

There was no mortality and ophthalmic changes related to NS during the administration period. No significant differences in the body weight of the animals treated with NS was observed relative to the controls during 13 weeks (Figure 3). For the males that received a dose of 500 and 2000 mg/kg/day, food consumptions were lower on 7 and 12 weeks and for the males that received 1000 mg/kg/day, food consumption was lower on 7, 9, 10 and 12 weeks when compared to the control group (Table 2).

**Table 2:** Food consumption of the rats during the study period

Groups (mg/kg/day)								
weeks	0		500		1000		2000	
	Male	Female	Male	Female	Male	Female	Male	Female
0	18.8 ± 1.0	13.9 ± 1.4	18.1 ± 1.2	14.1 ± 1.7	19.6 ± 0.3	13.3 ± 1.0	20.1 ± 0.9	13.1 ± 1.5
1	21.3 ± 0.8	15.5 ± 0.5	21.1 ± 1.0	15.2 ± 1.3	20.4 ± 0.9	14.3 ± 1.4	21.2 ± 1.1	13.9 ± 1.0
2	22.0 ± 1.2	12.6 ± 1.2	21.2 ± 0.4	13.5 ± 0.7	20.5 ± 1.3	12.9 ± 1.2	19.5 ± 1.1	12.8 ± 0.9
3	22.2 ± 1.5	12.4 ± 1.4	20.3 ± 0.4	10.8 ± 0.8	20.2 ± 1.1	11.8 ± 1.2	21.1 ± 2.1	11.6 ± 0.3
4	20.2 ± 1.7	13.3 ± 1.4	18.4 ± 1.0	12.9 ± 0.8	18.6 ± 0.9	13.1 ± 0.4	19.7 ± 1.1	11.7 ± 1.8
5	20.8 ± 1.3	14.1 ± 1.0	21.0 ± 0.6	13.9 ± 1.4	20.0 ± 0.7	13.3 ± 1.2	20.1 ± 0.7	13.9 ± 1.1
6	21.5 ± 1.2	13.6 ± 0.6	21.6 ± 0.9	14.6 ± 1.1	21.1 ± 2.1	14.5 ± 1.7	21.4 ± 1.2	13.8 ± 0.4
7	22.4 ± 0.7	15.3 ± 1.2	21.2 ± 1.1^*^	15.5 ± 1.0	20.7 ± 1.0^*^	15.2 ± 1.5	20.7 ± 0.7^*^	14.5 ± 1.6
8	22.0 ± 1.6	14.2 ± 0.4	20.9 ± 0.5	13.5 ± 1.4	20.4 ± 1.1	12.9 ± 1.0	21.0 ± 1.0	12.6 ± 0.7
9	23.0 ± 1.1	15.6 ± 1.2	22.1 ± 1.3	15.3 ± 1.0	20.3 ± 0.9^**^	14.1 ± 1.2	22.1 ± 0.7	14.7 ± 1.4
10	20.2 ± 1.3	13.6 ± 0.6	18.6 ± 1.3	12.5 ± 1.2	17.4 ± 1.8^*^	12.8 ± 1.0	19.2 ± 1.1	12.1 ± 1.5
11	20.5 ± 0.8	12.9 ± 1.2	19.0 ± 1.9	12.3 ± 1.2	19.0 ± 0.7	13.4 ± 0.4	20.0 ± 1.7	12.0 ± 1.0
12	20.5 ± 1.3	13.3 ± 0.9	18.7 ± 1.2^*^	13.5 ± 0.1	18.3 ± 0.8^**^	12.5 ± 0.8	19.1 ± 0.9^*^	12.8 ± 1.5
13	17.4 ± 1.8	12.4 ± 1.1	18.2 ± 0.4	12.7 ± 0.9	18.9 ± 1.0	12.5 ± 1.0	16.0 ± 2.8	12.1 ± 0.5
N	10	10	10	10	10	10	10	10

*P < 0.05, **P < 0.01 compared to the control group.

In the hematological examination, a significant increase in hemoglobin concentration distribution width (HDW) values was observed in males that received a dose of 500, 1000 and 2000 mg/kg/day. In addition, the red cell distribution width (RDW) values were higher in males treated with 500 and 2000 mg/kg/day (Table 3). Since these changes were not consistent between the males and females group, they were likely unrelated to the administration of NS.

**Table 3:** Hematological values following 13 weeks of daily oral administration of NS in male and female rats

Hematology values											
Dose (mg/kg)	Sex	WBC	RBC	HGB	HCT	MCV	MCH	MCHC	RDW	HDW	PLT
		(10^3^/μl)	(10^6^/μl)	(g/dl)	(%)	(fl)	(pg)	(g/μl)	(%)	(g/dl)	(10^3^/μl)
0	M	9.1 ± 1.1	9.2 ± 0.3	16.1 ± 0.5	51.3 ± 1.6	56.1 ± 1.3	17.6 ± 0.5	31.4 ± 0.4	12.0 ± 0.3	2.1 ± 0.1	1140 ± 98
	F	4.9 ± 1.6	7.8 ± 0.3	14.6 ± 0.4	47.0 ± 1.3	60.3 ± 1.1	18.7 ± 0.6	31.0 ± 0.7	10.8 ± 0.2	1.9 ± 0.1	1147 ± 106
500	M	8.2 ± 1.5	9.0 ± 0.2	16.0 ± 0.4	51.5 ± 0.9	57.2 ± 1.3	17.9 ± 0.5	31.2 ± 0.5	12.3 ± 0.2^*^	2.25 ± 0.1^**^	1066 ± 115
	F	4.7 ± 1.6	7.9 ± 0.2	14.5 ± 0.4	47.1 ± 1.3	59.4 ± 0.6	18.3 ± 0.4	30.8 ± 0.6	10.8 ± 0.4	2.0 ± 0.2	1111 ± 64
1000	M	9.1 ± 1.4	8.9 ± 0.3	15.8 ± 0.7	50.7 ± 1.7	56.7 ± 1.0	17.7 ± 0.5	31.3 ± 0.4	12.3 ± 0.4	2.2 ± 0.1^*^	1121 ± 45
	F	4.2 ± 2.1	7.9 ± 0.2	14.6 ± 0.3	47.4 ± 1.1	60.4 ± 1.7	18.5 ± 0.6	30.7 ± 0.3	10.9 ± 0.4	1.9 ± 0.2	1081 ± 77
2000	M	9.3 ± 2.1	9.3 ± 0.4	16.0 ± 0.5	51.6 ± 1.8	55.7 ± 2.0	17.3 ± 0.7	31.0 ± 0.4	12.4 ± 0.4^*^	2.2 ± 0.1^**^	1177 ± 143
	F	4.8 ± 1.8	7.7 ± 0.3	14.4 ± 0.5	17.0 ± 1.4	60.8 ± 1.0	18.6 ± 0.3	30.6 ± 0.3	10.8 ± 0.4	2.0 ± 0.2	1063 ± 113

HCT = Hematocrit, HDW = Hb conc. Distribution width, HGB = Hemoglobin concentration, MCH = Mean corpuscular hemoglobin, MCHC = Mean corpuscular hemoglobin concentration, MCV = Mean corpuscular volume, PLT = Platelet, RBC = Red blood cell, RDW = Red cell distribution width, WBC = White blood cell.

*P < 0.05, **P < 0.01 compared to the control group.

In the blood chemical examination, AST and ALT levels were higher in the all females that received NS treatment and the CPK level was lower in for both the male and female rats that received NS treatment. However, the differences were not significant (Table 4).

**Table 4:** Serum biochemical values following 13 weeks of daily oral administration of NS in male and female rats

Serum Chemistry Values											
Dose (mg/kg)	Sex	AST	ALT	ALP	BUN	CRE	GLU	CHO	PRO	CPK	ALB
		(IU/L)	(IU/L)	(IU/L)	(mg/dl)	(mg/dl)	(mg/dl)	(mg/dl)	(g/dl)	(IU/L)	(g/dl)
0	M	83.3 ± 15.0	40.4 ± 9.0	83.2 ± 14.2	16.2 ± 1.6	0.5 ± 0.1	132.2 ± 14.1	113.1 ± 15.7	6.2 ± 0.2	211.3 ± 129	3.0 ± 0.1
	F	85.7 ± 12.5	33.6 ± 7.8	74.3 ± 18.6	15.1 ± 1.3	0.6 ± 0.0	108.2 ± 6.9	95.2 ± 18.5	6.0 ± 0.4	186.8 ± 72.0	3.1 ± 0.2
500	M	80.0 ± 12.1	42.2 ± 5.4	90.6 ± 23.6	16.2 ± 3.0	0.5 ± 0.1	123.6 ± 12.1	108.0 ± 18.2	6.1 ± 0.1	173.1 ± 86.8	2.95 ± .01
	F	92.9 ± 13.1	33.4 ± 3.9	69.8 ± 12.4	16.4 ± 3.0	0.6 ± 0.1	114.4 ± 7.5	90.1 ± 12.8	6.1 ± 0.2	173.6 ± 74.8	3.1 ± 0.1
1000	M	84.1 ± 17.1	40.9 ± 6.1	92.1 ± 18.8	15.4 ± 1.6	0.5 ± 0.1	131.1 ± 17.0	103.1 ± 21.7	6.1 ± 0.2	157.8 ± 97.1	3.0 ± 0.1
	F	96.0 ± 21.0	35.0 ± 7.0	69.3 ± 16.9	16.0 ± 2.3	0.6 ± 0.1	107.5 ± 9.6	96.9 ± 20.3	6.2 ± 0.3	173.4 ± 87.4	3.2 ± 0.2
2000	M	83.1 ± 19.3	44.2 ± 12.4	84.0 ± 13.7	16.9 ± 3.6	0.5 ± 0.0	133.6 ± 12.6	111.4 ± 27.3	6.3 ± 0.1	122.3 ± 65.2	3.0 ± 0.1
	F	98.8 ± 22.5	35.8 ± 4.6	77.6 ± 14.2	16.4 ± 2.0	0.6 ± 0.1	112.8 ± 10.3	90.0 ± 20.1	6.0 ± 0.4	141.0 ± 61.2	3.1 ± 0.1

ALB = Albumin, ALP = Alkaline phosphatase, ALT = Alanine aminotransferase, AST = Aspartate aminotransferase, BUN = Blood urea nitrogen, CHO = Total cholesterol, CPK = Creatine phosphokinase, CRE = Creatinine, GLU = Glucose, PRO = Total protein.

**Figure 4:**
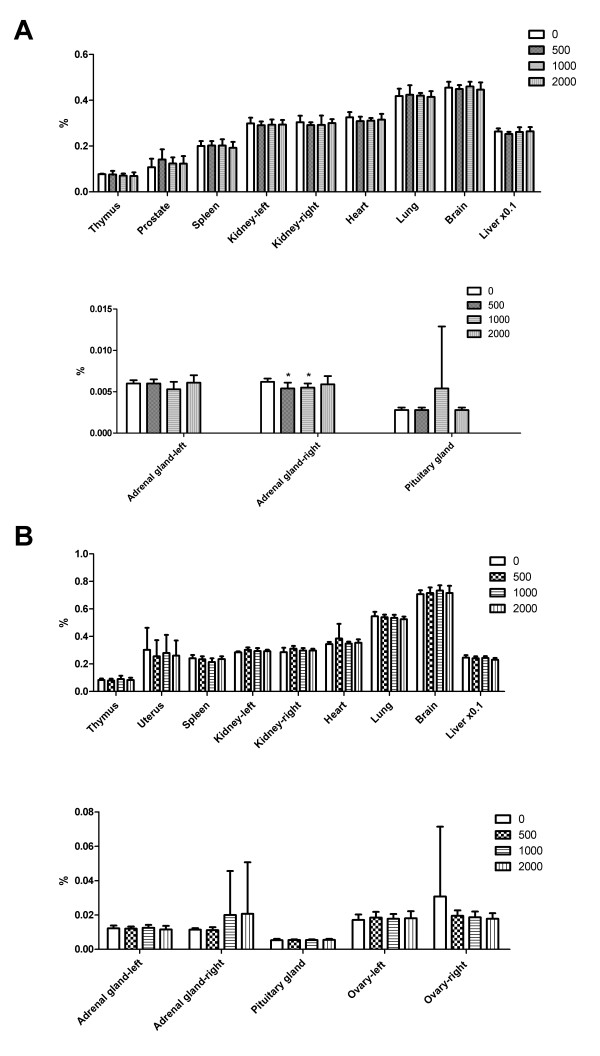
Percentage organ weight to body mass of male (A) and female (B) rats fed NS.

Figures 4 shows the organ-to-body mass ratios of animals at the end of the 13 week treatment. Except for the right adrenal glands in male rat that were administered the 500 and 1000 mg/kg dose, no abnormal changes were observed in organ mass with respect to body mass of NS fed rats in comparison with controls. Although the weight of the right adrenal glands was lower in male rat the received the 500 and 1000 mg/kg dose, the changes were not does-dependent nor sex matched. Thus, these changes were considered to be unrelated with NS treatment. When the gross pathology immediately after dissection in rats of all groups was examined, all animals were found to be uniformly healthy, lacking any apparent pathological abnormalities.

### 28- day dog does range finding (DRF) studies

In previous toxicity studies using rats, no toxicologically significant signs were observed up to a dose of 2000 mg/kg. Before commencing administration in the present study, one male and female, which were remnants after grouping, was provided one dose of 2000 and 1000 mg/kg NS and no toxicological signs were found during five days of observation. Therefore, in the present study, the high dose was set at 4000 mg/kg and three lower dose groups were 2000, 1000 and 500 mg/kg.

**Figure 5:**
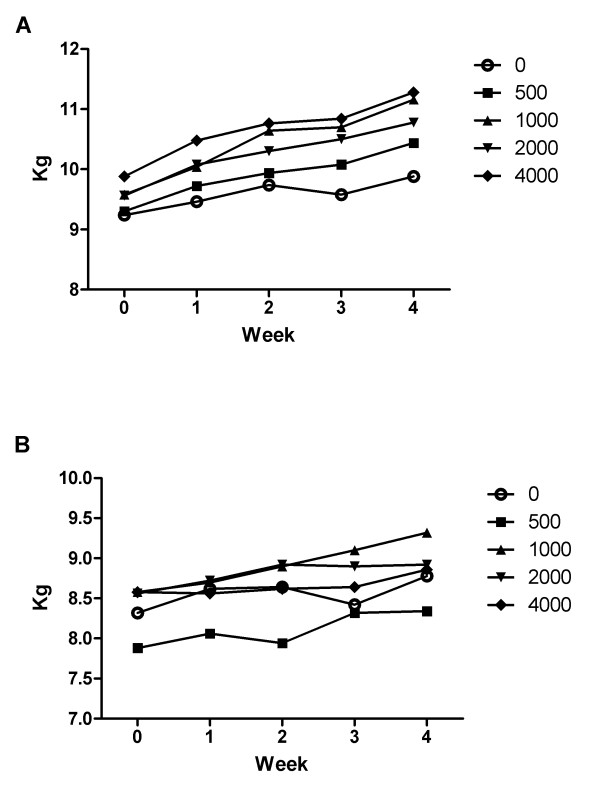
Body weight changes of male (A) and female (B) dogs given NS orally for 4 weeks.

There was no mortality related to NS after 28 days. Vomiting was observed in male rats that received a dose of 2000 mg/kg on the day of 9th administration. Feeding was sporadically observed in the female treated groups (Data not shown). In addition, no significant changes related to NS administration were observed (Figure 5).

In the urinalysis test, no unique changes related to NS administration were observed. Proteinuria was observed in males that received a dose of 0, 500 and 1000 mg/kg and in females that received 500, 1000 and 4000 mg/kg, and a low specific gravity (SG) of the urine was observed in all female groups. Furthermore, there was a positive reaction in the urine occult blood for males and females receiving a dose of 2000 mg/kg and 4000 mg/kg, respectively. In addition, a WBC reaction was observed in males that were treated with 0, 500, 2000 and 4000 mg/kg and females that recieved 2000 mg/kg (Table 5). However, these effects were not dose-dependent, not accompanied with other corresponding changes, and were observed before administration of NS. Therefore, these effects were not related to NS administration.

**Table 5:** Urin analysis in male and female dogs

**Groups (mg/kg/day) male**										
	0		500		1000		2000		4000	
	Before	After	Before	After	Before	After	Before	After	Before	After
GLU	+/−	+/−	-	-	-	-	-	-	-	-
BIL	1+	-	-	1+	-	-	1+	-	-	-
KET	-	-	-	-	-	-	-	-	-	-
SG	1.020	≤1.005	1.015	1.015	1.010	≥1.030	1.010	≤1.005	≤1.005	≤1.005
pH	7.0	≥9.0	8.0	8.0	8.0	7.0	8.5	≥9.0	≥9.0	8.5
PRO	1+	3+	1+	2+	3+	2+	1+	+/−	3+	+/−
URO	0.1	0.1	0.1	0.1	0.1	0.1	0.1	0.1	1.0	0.1
NIT	-	-	-	-	-	-	-	-	-	-
OB	-	-	-	-	-	-	2+	1+	-	1+
WBC	2+	2+	1+	2+	1+	+/−	2+	3+	2+	3+
**Groups (mg/kg/day) female**										
	0		500		1000		2000		4000	
	Before	After	Before	After	Before	After	Before	After	Before	After
GLU	-	-	-	-	-	-	-	-	-	-
BIL	-	-	-	-	-	-	-	-	-	-
KET	-	-	-	-	-	-	-	-	-	-
SG	≤1.005	≤1.005	≥1.030	≤1.005	≥1.030	≤1.005	≥1.030	≤1.005	≥1.030	≤1.005
pH	8.5	≥9.0	6.5	≥9.0	6.5	8.5	6.5	8.0	6.5	≥9.0
PRO	-	+/−	2+	1+	2+	3+	1+	-	2+	2+
URO	0.1	0.1	0.1	0.1	1.0	0.1	0.1	0.1	0.1	0.1
NIT	-	-	-	-	-	-	-	-	-	-
OB	-	-	-	-	-	-	-	-	-	2+
WBC	-	+/−	-	-	-	+/−	-	1+	+/−	+/−

GLU = Glucose, BIL = bilirubin, KET = ketone body, SG = specific gravity, OB = occult blood, pH, PRO = protein, URO = urobilinogen, NIT = nitrite and WBC = leukocyte.

**Figure 6:**
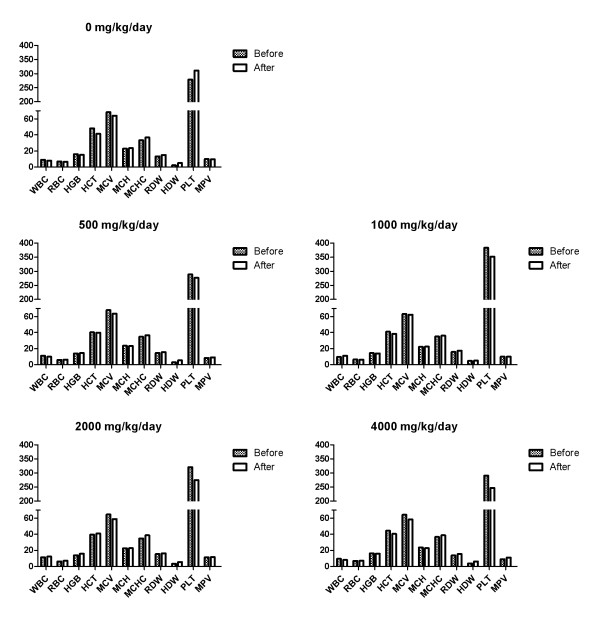
**Hematological data of male dogs fed with NS for 28 Days.** HCT = Hematocrit, HGB = Hemoglobin concentration, MCH = Mean corpuscular hemoglobin, MCHC = Mean corpuscular hemoglobin concentration, MCV = Mean corpuscular volume, PLT = Platelet, RBC = Red blood cell, WBC = White blood cell.

**Figure 7:**
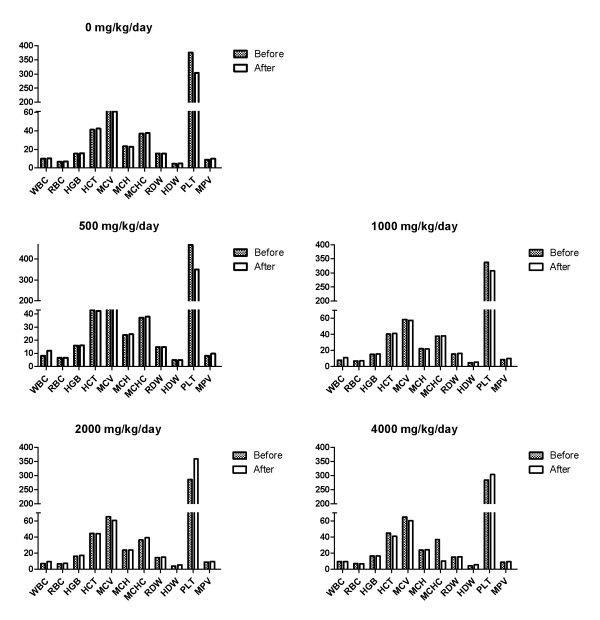
**Hematological data of female dogs fed with NS for 28 Days.** HCT = Hematocrit, HGB = Hemoglobin concentration, MCH = Mean corpuscular hemoglobin, MCHC = Mean corpuscular hemoglobin concentration, MCV = Mean corpuscular volume, PLT = Platelet, RBC = Red blood cell, WBC = White blood cell

**Figure 8:**
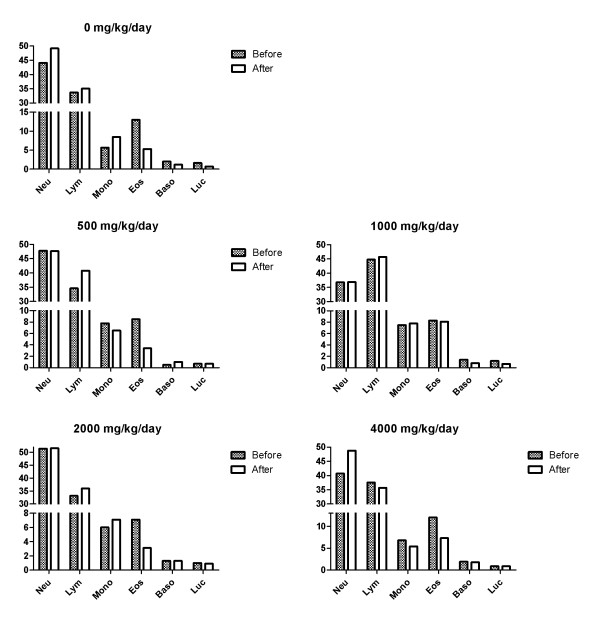
**Differential leukocyte counts in male dogs.** NEU = Neutrophil, LYM = Lymphocyte, MONO = Monocyte, EOS = Eosinophil, BASO = Basophil, LUC = Large unstained cells

**Figure 9:**
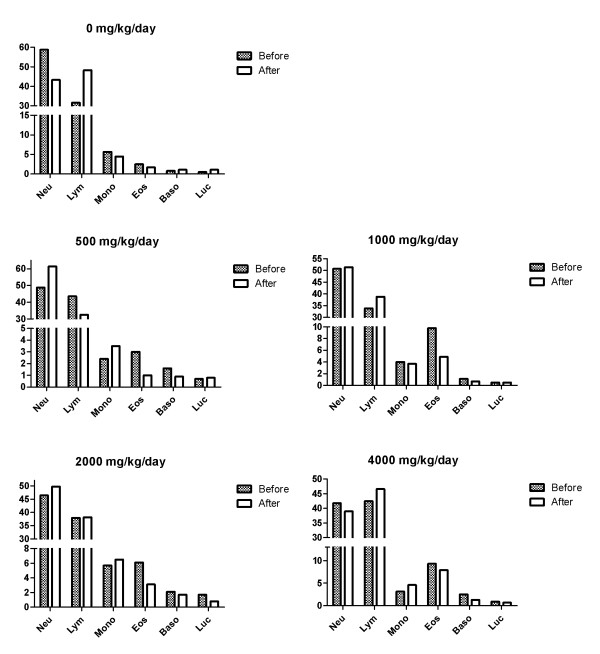
**Differential leukocyte counts in female dogs.** NEU = Neutrophil, LYM = Lymphocyte, MONO = Monocyte, EOS = Eosinophil, BASO = Basophil, LUC = Large unstained cells.

In the hematological test, toxicologically significant changes related to NS administration were observed (Figures 6 and 7). In addition, there were no toxicologically significant changes related to NS administration in the serum biochemical data of male and female dogs fed with NS for 28 Days (Tables 6 and 7). The result of the hematological examinations and differential leukocyte counts, which were examined with blood taken at the necropsy, were similar between all the groups (Figures 8 and 9).

**Table 6:** Serum biochemical values in male dogs

Groups (mg/kg/day) male										
	0		500		1000		2000		4000	
	Beforentry	After	Before	After	Before	After	Before	After	Before	After
AST	29.6	35.7	29.2	32.6	25.6	26.3	25.9	28.7	26.1	37.8
ALT	28.1	33.6	30.6	33.8	26.9	32.4	30.8	38.8	23.5	32
ALP	111.3	116.1	143.4	130.6	185.5	227.2	167.7	145.9	104.7	143
BUN	11.8	17.8	11.6	18.7	14.6	16.1	10.4	14.1	12.6	17.3
CRE	0.57	0.75	0.49	0.77	0.64	0.66	0.64	0.65	0.71	0.75
GLU	97.4	86.8	98.4	92.6	97.7	92.1	91.8	87.2	101.0	102.1
CHO	199	140	207	170	136	115	181	154	156	151
PRO	5.5	5.76	5.08	5.41	5.47	5.65	5.18	5.48	5.42	5.82
CPK	170	187	233	293	174	157	185	206	136	220
ALB	2.93	3.13	2.61	2.91	2.88	3.16	2.7	2.98	2.64	3.2
T-BIL	0.24	0.26	0.21	0.22	0.23	0.23	0.20	0.22	0.24	0.21
TG	29	42	40	62	30	33	48	50	35	43
IP	6.95	6.43	6.02	6.21	7.06	6.97	6.95	6.42	6.45	5.66
Ca	11.09	10.77	10.64	11.3	10.86	11.19	10.43	11.14	10.22	11.02
A/G	1.14	1.19	1.06	1.16	1.11	1.27	1.09	1.19	0.95	1.22
Na	150	147	150	146	153	147	148	144	151	146
K	4.95	4.73	4.88	4.85	5.06	4.99	5.53	5.48	4.75	4.83
Cl	111	116	112	116	115	115	110	114	111	115

A/G ratio = Albumin/globulin ratio, ALB = Albumin, ALP = Alkaline phosphatase, ALT = Alanine aminotransferase, AST = Aspartate aminotransferase, T-BIL = Total bilirubin, BUN = Blood urea nitrogen, Ca = Calcium, CHO = Total cholesterol, Cl = Chloride, CPK = Creatine phosphokinase, CRE = Creatinine, GLU = Glucose, IP = Inorganic phosphorus, K = Potassium, Na = Sodium, PRO = Total protein, TG = Triglyceride.

**Table 7:** Serum biochemical values in female dogs

Groups (mg/kg/day) female										
	0		500		1000		2000		4000	
	Before	After	Before	After	Before	After	Before	After	Before	After
AST	26.7	36.1	32.1	35.1	30.3	39.7	22.5	28.6	26	35.1
ALT	30.9	91.8	30.2	28.1	20.2	24.8	25	30.1	37.2	42.9
ALP	107.6	119.5	127.6	155.9	95.6	87.1	115	88.5	115.1	102.9
BUN	10.3	13.1	17.1	17.4	13.4	14.7	14.9	17.6	10.4	14.1
CRE	0.7	0.74	0.62	0.76	0.7	0.73	0.7	0.66	0.7	0.7
GLU	99.8	107.3	112.8	109.8	108.4	106.1	95.4	86.4	107.9	107.4
CHO	181	169	167	145	149	110	234	172	197	164
PRO	5.42	5.54	5.27	5.56	5.41	5.71	5.53	5.76	5.49	6.04
CPK	177	192	206	188	142	207	136	164	217	245
ALB	2.99	3.20	2.98	3.21	2.94	3.19	2.88	3.20	3.06	3.16
T-BIL	0.26	0.28	0.24	0.23	0.25	0.26	0.25	0.27	0.25	0.25
TG	29	31	34	29	42	39	29	40	34	46
IP	6.2	5.71	6.45	6.60	6.57	5.94	6.36	6.73	6.2	5.65
Ca	10.53	11.06	10.31	11.28	10.74	11.50	10.65	11.16	10.23	10.93
A/G	1.23	1.37	1.30	1.37	1.19	1.27	1.09	1.25	1.26	1.1
Na	151	144	150	147	151	148	151	148	151	147
K	5.31	4.74	5.60	4.77	4.73	4.59	4.74	4.46	4.73	4.73
Cl	113	115	112	115	113	116	115	115	114	117

A/G ratio = Albumin/globulin ratio, ALB = Albumin, ALP = Alkaline phosphatase, ALT = Alanine aminotransferase, AST = Aspartate aminotransferase, T-BIL = Total bilirubin, BUN = Blood urea nitrogen, Ca = Calcium, CHO = Total cholesterol, Cl = Chloride, CPK = Creatine phosphokinase, CRE = Creatinine, GLU = Glucose, IP = Inorganic phosphorus, K = Potassium, Na = Sodium, PRO = Total protein, TG = Triglyceride.

**Figure 10:**
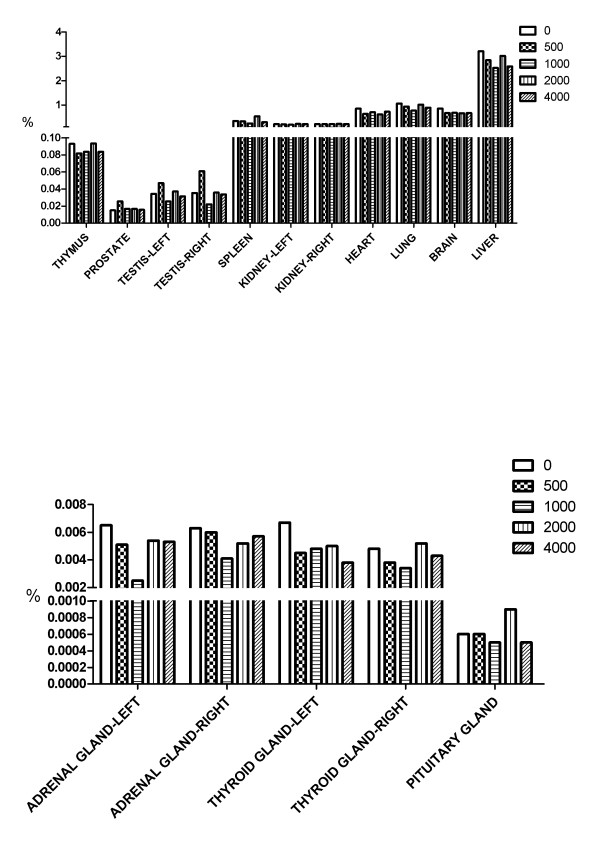
**Effect of NS orally administered for 28 days on organ-body weight indices in male Beagle dogs.** The organ-body weight index was calculated using the following formula: Organ-body weight index (%) = Wet organ weight/Body weight × 100%.

**Figure 11:**
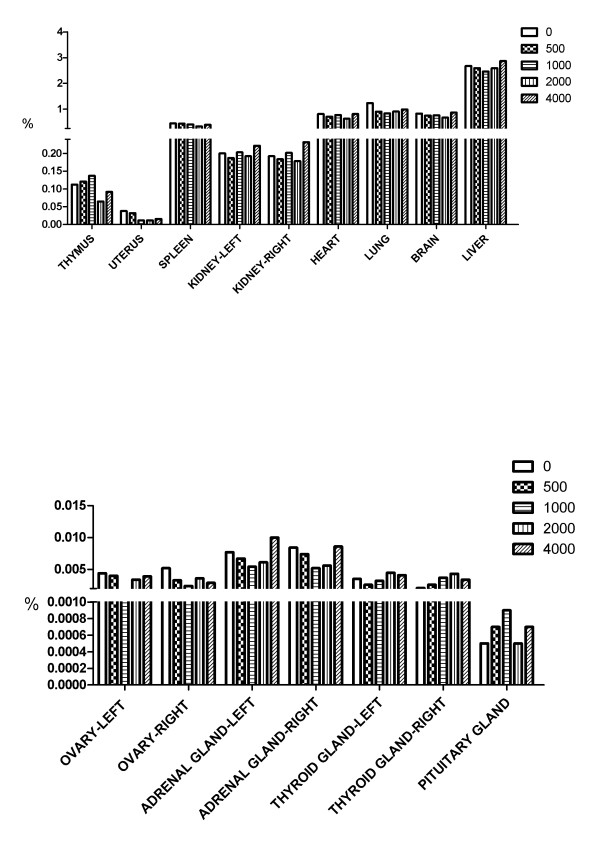
**Effect of NS orally administered for 28 days on organ-body weight indices in female Beagle dogs.** The organ-body weight index was calculated using the following formula: Organ-body weight index (%) = Wet organ weight/Body weight × 100%.

An autopsy study was carried out after the animals were treated for 28 days. The organs, including heart, liver, spleen, lung, kidney, adrenal gland, thymus, thyroid gland, brain, uterus, ovary and testis were carefully examined. No noticeable pathologic changes were observed by the naked eye. Moreover, no significant differences in the organ-bodyweight indices of the organs mentioned above were found (Figures 10 and 11). After 28 days of NS treatment, histopathological investigations were carried out. No pathological changes were observed in any organs of these animals (Data not shown).

## Discussion

Most components of *Nelumbo nucifera* such as the root, leaf, seed, and stem are edible, and they have been used as side dishes or tea in eastern Asia. Although the seed of *Nelumbo nucifera* has been used to treat various psychological disorders in traditional medicine, its effects and mechanisms are still largely unknown. However, many recent reports have examined its effects in various disease models. A review on this plant by Mukherjee et al. [16] stated that various parts of this plant display potential therapeutic activity, such as hypoglycemic, antidiarrheal, antimicrobial, diuretic, antipyretic as well as anti-inflammatory activities of its rhizomes extracts [17,18]. Hepatoprotective [19], antiproliferative [20], anti-inflammatory as well as antioxidant [21] activities of its seed extracts have also been reported. In addition, many studies regarding the anti-depressive effects and mechanism of Nelumbinis Semen (NS) have recently been performed. Kang et al. reported that NS reverses a decrease in 5-HT1A receptor binding and hippocampal 5-HT release induced by chronic mild stress, which is a depression-like symptom [1,7]. These studies suggested that Nelumbinis Semen increases serotonin levels, which are normally lower during depression, and enhances central serotonergic transmission. Thus, NS may be used for the treatment of depression. Sugimoto et al., also showed that neferine, an alkaloid of NS, has antidepressant-like effects in mice similar to typical antidepressants and that these effects are mediated by the 5-HT1A receptor [22].

In this study, we evaluated the anti-depressive effects of NS based on behavior tests in a forced swimming test and a chronic mild stress model. When we evaluated the maximal effective dose in a forced swimming test, 400 mg/kg of NS was shown the most shortened immobility times. When we compared NS with other well known anti-depressants such as fluoxetine and St. John’s Wort, NS showed equal or higher anti-depressive effects compared with these commercially available anti-depressants. Even if NS is effective to treat depression, its toxicity must be evaluated before it can be utilized as a new drug.

Thus, in this study, we carried out NS toxicity studies in rats and dogs. In the rat toxicity studies, NS was administered orally at dose levels of 500, 1000, and 2000 mg/kg/day. In these experiments, NS caused no changes that could be considered toxicologically significant. In the serum biochemical analysis, ALT and AST levels decreased in only NS fed female rats. However, these changes were not significant and no correlated changes were observed in necropsy findings and organ weight. Significant hematological changes were observed in male rats that received doses of 500 and 2000 mg/kg/day, including statistically significant increases in RDW and HDW in all NS fed rats. However, these changes were not consistent across the different genders and no changes in leukocytes differentiation were observed. Consequently, repeated oral administration of NS to rats for 13 weeks resulted in no toxicological changes in any of the examinations, namely mortality, clinical signs, body weight changes, food and water consumption, urinalysis, hematology and serum biochemistry examinations, necropsy findings and relative organ weights, at the doses tested. Thus, under the present experimental conditions, the NOAEL of NS was assumed to be 2000 mg/kg/day for both males and females.

Based on subchronic toxicity results in SD rats, we also wanted to investigate the toxicity of NS by repeated oral administration in Beagle dogs. NS was administered to male and female beagle dogs at dose levels of 500, 1000, 2000 and 4000 mg/kg. There were no consistent and dose-related changes that could be attributed to NS administration in regards to mortality, body weight, food intake, ophthalmoscopy, urinalysis, hematology, serum biochemistry, necropsy finding and organ weights. Vomiting was observed only in the male group and may not have been caused be administration of NS since the vomiting could have been induced by gastro-intestinal stimuli, which is common in dogs [23]. The sporadically feeding observed in female groups was not dose-dependent and this has also been observed frequently in dogs. Therefore, these effects were most likely not caused by NS administration. In the urinalysis, a low SG, positive reaction for proteins, occult blood and WBC were observed. However, these effects were not dose-dependent, not accompanied by other corresponding changes and were also observed before NS administration. Therefore, these effects were not related to NS treatemtn. Based on the results of this study, no systemic and toxicologically significant change related to NS administration were observed in the 4-week repeated dose experiment and the NOAEL (No observed adverse effect level) was determined to be 4000 mg/kg/day.

## Conclusion

Based on our observations, NS significantly reversed depressive symptoms under chronic mild stress. In addition, in the safety evaluation studies, NS was shown to be safe up to a dose of 4000 mg/kg/day over 13 weeks of administration in rats and up to 2000 mg/kg/day over 4 weeks of administration in dogs. The results presented here provide a foundation for further clinical research and demonstrate the potential of using NS as a new herbal drug.

## Competing interests

The authors declare that they have no competing interests.

## Authors’ contributions

H.C, H. L and I. S mainly performed the animal experiment, analyzed the data. H. B supervised the project and wrote the final paper. All authors read and approved the final manuscript.

## Pre-publication history

The pre-publication history for this paper can be accessed here:

http://www.biomedcentral.com/1472-6882/12/68/prepub
